# Supplementary material for: Validity of cerebrovascular ICD-9-CM codes in healthcare administrative databases. The Umbria Data-Value Project
Source: PLoS One. 2020 Jan 9;15(1):e0227653. doi: 10.1371/journal.pone.0227653 (PMC6952250; doi:10.1371/journal.pone.0227653)
Supplement: S1 Dataset — (PDF) [file pone.0227653.s002.pdf]

Subarachnoid hemorrhage

Number Clinical Chart	Hospital	Gender	Patient's age on admission	Primary Diagnosis (ICD-9)	Presence of neurological signs, symptoms or coma	Positive CT or MRI	Other positive instrumental test	Validation
Chart_1	Hospital_1	M	77	430	1	1	1	1
Chart_2	Hospital_1	F	60	430	1	1	0	1
Chart_3	Hospital_1	M	60	430	1	1	0	1
Chart_4	Hospital_1	F	62	430	1	1	1	1
Chart_5	Hospital_1	F	76	430	1	2	2	0
Chart_6	Hospital_1	F	68	430	1	1	1	1
Chart_7	Hospital_1	F	52	430	1	1	2	1
Chart_8	Hospital_1	M	80	430	1	1	2	1
Chart_9	Hospital_1	M	84	430	1	1	2	1
Chart_10	Hospital_1	F	57	430	1	1	1	1
Chart_11	Hospital_1	F	73	430	1	1	1	1
Chart_12	Hospital_1	F	86	430	1	1	1	1
Chart_13	Hospital_1	M	75	430	1	1	0	1
Chart_14	Hospital_1	F	92	430	1	1	2	1
Chart_15	Hospital_1	F	71	430	1	1	1	1
Chart_16	Hospital_1	F	45	430	1	1	1	1
Chart_17	Hospital_1	F	63	430	1	1	0	1
Chart_18	Hospital_1	M	58	430	1	1	1	1
Chart_19	Hospital_1	M	72	430	1	1	0	1
Chart_20	Hospital_1	F	69	430	1	1	1	1
Chart_21	Hospital_1	M	65	430	1	1	2	1
Chart_22	Hospital_1	M	43	430	1	1	1	1
Chart_23	Hospital_1	F	64	430	1	1	1	1
Chart_24	Hospital_1	F	70	430	1	1	2	1
Chart_25	Hospital_1	F	60	430	1	1	1	1
Chart_26	Hospital_1	M	81	430	1	1	2	1
Chart_27	Hospital_1	F	36	430	1	1	2	1
Chart_28	Hospital_1	M	48	430	1	1	2	1
Chart_29	Hospital_1	F	53	430	1	1	1	1
Chart_30	Hospital_1	F	65	430	1	1	2	1
Chart_31	Hospital_1	F	51	430	1	1	1	1
Chart_32	Hospital_1	F	49	430	1	1	1	1

Number Clinical Chart	Hospital	Gender	Patient's age on admission	Primary Diagnosis (ICD-9)	Presence of neurological signs, symptoms or coma	Positive CT or MRI	Other positive instrumental test	Validation
Chart_33	Hospital_1	F	43	430	1	1	1	1
Chart_34	Hospital_1	F	--	430	n.a.	n.a.	n.a.	n.a.
Chart_35	Hospital_1	F	81	430	1	2	1	1
Chart_36	Hospital_1	M	47	430	1	1	1	1
Chart_37	Hospital_1	F	39	430	1	1	1	1
Chart_38	Hospital_1	F	84	430	1	1	2	1
Chart_39	Hospital_1	M	31	430	1	1	2	1
Chart_40	Hospital_1	M	33	430	1	1	1	1
Chart_41	Hospital_1	F	86	430	1	1	2	1
Chart_42	Hospital_1	M	67	430	1	1	1	1
Chart_43	Hospital_1	F	51	430	1	1	1	1
Chart_44	Hospital_1	F	84	430	1	1	1	1
Chart_45	Hospital_1	M	46	430	1	1	2	1
Chart_46	Hospital_1	M	66	430	1	1	2	1
Chart_47	Hospital_1	F	90	430	1	1	2	1
Chart_48	Hospital_1	M	36	430	1	1	1	1
Chart_49	Hospital_1	F	67	430	1	1	1	1
Chart_50	Hospital_1	M	44	430	1	1	2	1
Chart_51	Hospital_1	M	43	430	1	1	2	1
Chart_52	Hospital_1	F	75	430	1	1	2	1
Chart_53	Hospital_1	F	78	430	1	1	1	1
Chart_54	Hospital_1	F	80	430	1	1	1	1
Chart_55	Hospital_1	F	67	430	1	1	1	1
Chart_56	Hospital_1	F	61	430	1	1	1	1
Chart_57	Hospital_1	M	78	430	1	1	2	1
Chart_58	Hospital_1	F	76	430	1	1	1	1
Chart_59	Hospital_1	M	81	430	1	1	2	1
Chart_60	Hospital_2	F	44	430	1	1	1	1
Chart_61	Hospital_2	M	52	430	1	1	1	1
Chart_62	Hospital_2	F	85	430	1	1	2	1
Chart_63	Hospital_2	F	46	430	1	1	1	1
Chart_64	Hospital_2	M	85	430	1	1	2	1
Chart_65	Hospital_2	F	80	430	1	1	1	1
Chart_66	Hospital_2	F	65	430	1	1	2	1

Number Clinical Chart	Hospital	Gender	Patient's age on admission	Primary Diagnosis (ICD-9)	Presence of neurological signs, symptoms or coma	Positive CT or MRI	Other positive instrumental test	Validation
Chart_67	Hospital_2	M	36	430	1	1	1	1
Chart_68	Hospital_2	M	50	430	1	1	2	1
Chart_69	Hospital_2	M	72	430	1	1	2	1
Chart_70	Hospital_2	M	50	430	1	1	1	1
Chart_71	Hospital_2	F	33	430	1	1	1	1
Chart_72	Hospital_2	M	58	430	1	1	2	1
Chart_73	Hospital_2	F	76	430	1	1	1	1
Chart_74	Hospital_2	F	72	430	1	1	1	1
Chart_75	Hospital_2	M	50	430	1	1	2	1
Chart_76	Hospital_2	F	72	430	1	1	2	1
Chart_77	Hospital_2	F	78	430	1	1	1	1
Chart_78	Hospital_2	F	72	430	1	1	2	1
Chart_79	Hospital_2	F	71	430	1	1	1	1
Chart_80	Hospital_2	M	62	430	1	1	1	1
Chart_81	Hospital_2	M	85	430	1	1	2	1
Chart_82	Hospital_2	F	56	430	1	1	1	1
Chart_83	Hospital_2	M	88	430	1	1	2	1
Chart_84	Hospital_2	M	80	430	1	1	2	1
Chart_85	Hospital_2	F	74	430	1	2	1	1
Chart_86	Hospital_2	M	70	430	1	1	1	1
Chart_87	Hospital_2	M	84	430	1	1	2	1
Chart_88	Hospital_2	F	34	430	1	1	1	1
Chart_89	Hospital_2	M	58	430	1	1	2	1
Chart_90	Hospital_2	F	72	430	1	1	1	1
Chart_91	Hospital_2	M	68	430	1	1	2	1
Chart_92	Hospital_2	F	86	430	1	1	2	1
Chart_93	Hospital_2	F	48	430	1	1	1	1
Chart_94	Hospital_2	M	62	430	1	1	0	1
Chart_95	Hospital_2	M	96	430	1	1	2	1
Chart_96	Hospital_2	F	90	430	1	1	2	1
Chart_97	Hospital_2	M	44	430	1	1	2	1
Chart_98	Hospital_2	M	89	430	1	1	2	1
Chart_99	Hospital_2	F	62	430	1	1	1	1
Chart_100	Hospital_3	F	83	430	1	1	2	1

Number Clinical Chart	Hospital	Gender	Patient's age on admission	Primary Diagnosis (ICD-9)	Presence of neurological signs, symptoms or coma	Positive CT or MRI	Other positive instrumental test	Validation
Chart_101	Hospital_3	F	98	430	1	2	2	0
Chart_102	Hospital_3	M	87	430	1	2	2	0
Chart_103	Hospital_3	M	88	430	1	1	2	1
Chart_104	Hospital_3	M	77	430	1	1	2	1
Chart_105	Hospital_3	M	76	430	1	1	2	1
Chart_106	Hospital_4	M	44	430	1	1	0	1
Chart_107	Hospital_4	F	74	430	1	1	2	1
Chart_108	Hospital_4	F	82	430	1	1	2	1
Chart_109	Hospital_4	F	92	430	1	1	2	1
Chart_110	Hospital_4	F	89	430	1	1	0	1
Chart_111	Hospital_4	M	76	430	1	1	2	1
Chart_112	Hospital_4	F	90	430	1	1	2	1
Chart_113	Hospital_4	M	62	430	1	1	2	1
Chart_114	Hospital_5	F	77	430	1	1	2	1
Chart_115	Hospital_5	F	95	430	1	1	2	1
Chart_116	Hospital_5	M	76	430	1	1	2	1
Chart_117	Hospital_5	F	81	430	1	1	2	1
Chart_118	Hospital_5	M	81	430	1	1	2	1
Chart_119	Hospital_5	F	80	430	1	1	2	1
Chart_120	Hospital_5	F	87	430	1	1	1	1
Chart_121	Hospital_6	F	80	430	1	1	2	1
Chart_122	Hospital_6	F	66	430	1	1	0	1
Chart_123	Hospital_7	M	96	430	1	1	2	1
Chart_124	Hospital_7	F	84	430	1	1	2	1
Chart_125	Hospital_7	M	84	430	1	1	2	1
Chart_126	Hospital_7	F	86	430	1	1	2	1
Chart_127	Hospital_7	F	64	430	1	1	2	1
Chart_128	Hospital_7	F	92	430	1	1	2	1
Chart_129	Hospital_7	F	88	430	1	1	2	1
Chart_130	Hospital_7	M	82	430	1	1	2	1

Legend: 0=no; 1=yes; 2=not reported; n.a.=clinical chart not available

Intracerebral haemorrhage

Number Clinical Chart	Hospital	Gender	Patient's age on admission	Primary Diagnosis (ICD-9)	Presence of neurological signs, symptoms or coma	Positive CT or MRI	Other positive instrumental test	Validation
Chart_1	Hospital_1	M	52	431	1	1	0	1
Chart_2	Hospital_1	M	77	431	1	1	2	1
Chart_3	Hospital_1	M	87	431	1	1	2	1
Chart_4	Hospital_1	F	89	431	1	1	2	1
Chart_5	Hospital_1	M	37	431	1	1	0	1
Chart_6	Hospital_1	M	79	431	1	1	2	1
Chart_7	Hospital_1	M	66	431	1	1	2	1
Chart_8	Hospital_1	F	75	431	1	1	2	1
Chart_9	Hospital_1	F	88	431	1	1	2	1
Chart_10	Hospital_1	M	82	431	1	1	2	1
Chart_11	Hospital_1	M	67	431	1	1	2	1
Chart_12	Hospital_1	F	78	431	1	1	2	1
Chart_13	Hospital_1	F	76	431	1	1	2	1
Chart_14	Hospital_1	F	66	431	1	1	1	1
Chart_15	Hospital_1	F	60	431	1	1	2	1
Chart_16	Hospital_1	F	75	431	1	1	2	1
Chart_17	Hospital_1	M	69	431	1	1	2	1
Chart_18	Hospital_1	M	82	431	1	1	1	1
Chart_19	Hospital_1	F	89	431	1	1	2	1
Chart_20	Hospital_1	F	88	431	1	1	2	1
Chart_21	Hospital_1	M	90	431	1	1	1	1
Chart_22	Hospital_1	F	44	431	1	1	2	1
Chart_23	Hospital_1	M	--	431	n.a.	n.a.	n.a.	n.a.
Chart_24	Hospital_1	F	80	431	1	1	2	1
Chart_25	Hospital_1	F	62	431	1	1	2	1
Chart_26	Hospital_1	M	74	431	1	1	2	1
Chart_27	Hospital_1	M	78	431	1	1	2	1
Chart_28	Hospital_1	M	--	431	n.a.	n.a.	n.a.	n.a.
Chart_29	Hospital_1	F	84	431	1	1	2	1
Chart_30	Hospital_1	M	103	431	1	1	2	1
Chart_31	Hospital_1	F	43	431	1	1	1	1
Chart_32	Hospital_1	M	75	431	1	1	2	1

Number Clinical Chart	Hospital	Gender	Patient's age on admission	Primary Diagnosis (ICD-9)	Presence of neurological signs, symptoms or coma	Positive CT or MRI	Other positive instrumental test	Validation
Chart_33	Hospital_1	M	84	431	1	1	2	1
Chart_34	Hospital_1	F	87	431	1	1	2	1
Chart_35	Hospital_1	M	79	431	1	1	2	1
Chart_36	Hospital_1	M	30	431	1	1	1	1
Chart_37	Hospital_1	M	79	431	1	1	0	1
Chart_38	Hospital_1	M	83	431	1	1	2	1
Chart_39	Hospital_1	M	80	431	1	1	1	1
Chart_40	Hospital_1	F	55	431	1	1	2	1
Chart_41	Hospital_1	F	46	431	1	1	0	1
Chart_42	Hospital_1	F	85	431	1	1	2	1
Chart_43	Hospital_1	M	--	431	n.a.	n.a.	n.a.	n.a.
Chart_44	Hospital_1	M	82	431	1	1	2	1
Chart_45	Hospital_1	F	69	431	1	1	2	1
Chart_46	Hospital_1	F	68	431	1	1	2	1
Chart_47	Hospital_1	M	46	431	1	1	1	1
Chart_48	Hospital_1	F	78	431	1	1	1	1
Chart_49	Hospital_1	M	58	431	1	1	2	1
Chart_50	Hospital_2	F	72	431	1	1	2	1
Chart_51	Hospital_2	F	81	431	1	1	0	1
Chart_52	Hospital_2	M	90	431	1	1	2	1
Chart_53	Hospital_2	M	71	431	1	1	0	1
Chart_54	Hospital_2	M	75	431	1	1	2	1
Chart_55	Hospital_2	F	80	431	1	1	2	1
Chart_56	Hospital_2	F	52	431	1	1	0	1
Chart_57	Hospital_2	F	85	431	1	1	2	1
Chart_58	Hospital_2	F	89	431	1	1	2	1
Chart_59	Hospital_2	M	65	431	1	1	2	1
Chart_60	Hospital_2	M	80	431	1	1	2	1
Chart_61	Hospital_2	F	76	431	1	1	2	1
Chart_62	Hospital_2	F	71	431	1	1	2	1
Chart_63	Hospital_2	F	82	431	1	1	2	1
Chart_64	Hospital_2	M	75	431	1	1	2	1
Chart_65	Hospital_2	M	57	431	1	1	2	1
Chart_66	Hospital_2	M	74	431	1	1	2	1

Number Clinical Chart	Hospital	Gender	Patient's age on admission	Primary Diagnosis (ICD-9)	Presence of neurological signs, symptoms or coma	Positive CT or MRI	Other positive instrumental test	Validation
Chart_67	Hospital_2	M	71	431	1	1	2	1
Chart_68	Hospital_2	M	75	431	1	1	2	1
Chart_69	Hospital_2	M	76	431	1	1	2	1
Chart_70	Hospital_2	F	83	431	1	1	2	1
Chart_71	Hospital_2	M	57	431	1	1	2	1
Chart_72	Hospital_2	M	72	431	1	1	2	1
Chart_73	Hospital_2	F	74	431	1	1	1	1
Chart_74	Hospital_2	M	77	431	1	1	2	1
Chart_75	Hospital_2	M	82	431	1	1	2	1
Chart_76	Hospital_2	F	70	431	1	1	2	1
Chart_77	Hospital_2	F	83	431	1	1	1	1
Chart_78	Hospital_2	M	88	431	1	2	2	0
Chart_79	Hospital_2	M	80	431	1	1	2	1
Chart_80	Hospital_2	F	81	431	1	1	2	1
Chart_81	Hospital_2	F	76	431	1	1	2	1
Chart_82	Hospital_2	F	90	431	1	1	2	1
Chart_83	Hospital_2	M	57	431	1	1	1	1
Chart_84	Hospital_2	F	83	431	1	1	2	1
Chart_85	Hospital_2	F	89	431	1	1	2	1
Chart_86	Hospital_2	F	48	431	1	1	2	1
Chart_87	Hospital_2	F	87	431	1	1	2	1
Chart_88	Hospital_3	F	80	431	1	1	2	1
Chart_89	Hospital_3	M	62	431	1	1	0	1
Chart_90	Hospital_3	F	88	431	1	1	1	1
Chart_91	Hospital_3	M	75	431	1	1	2	1
Chart_92	Hospital_3	F	90	431	1	1	2	1
Chart_93	Hospital_3	M	82	431	1	1	2	1
Chart_94	Hospital_3	M	83	431	1	1	0	1
Chart_95	Hospital_3	F	83	431	1	1	2	1
Chart_96	Hospital_3	M	84	431	1	1	2	1
Chart_97	Hospital_3	F	78	431	1	1	2	1
Chart_98	Hospital_4	F	--	431	n.a.	n.a.	n.a.	n.a.
Chart_99	Hospital_4	F	88	431	1	1	2	1
Chart_100	Hospital_4	F	80	431	1	1	2	1

Number Clinical Chart	Hospital	Gender	Patient's age on admission	Primary Diagnosis (ICD-9)	Presence of neurological signs, symptoms or coma	Positive CT or MRI	Other positive instrumental test	Validation
Chart_101	Hospital_4	M	68	431	1	1	2	1
Chart_102	Hospital_4	F	82	431	1	1	2	1
Chart_103	Hospital_4	F	52	431	1	1	2	1
Chart_104	Hospital_4	M	80	431	1	1	2	1
Chart_105	Hospital_5	M	74	431	1	1	2	1
Chart_106	Hospital_5	F	86	431	1	1	2	1
Chart_107	Hospital_5	F	79	431	1	1	2	1
Chart_108	Hospital_5	F	--	431	n.a.	n.a.	n.a.	n.a.
Chart_109	Hospital_5	M	61	431	1	1	2	1
Chart_110	Hospital_5	F	54	431	1	1	2	1
Chart_111	Hospital_5	F	91	431	1	1	2	1
Chart_112	Hospital_5	M	80	431	1	1	2	1
Chart_113	Hospital_5	M	76	431	1	1	1	1
Chart_114	Hospital_5	F	90	431	1	1	2	1
Chart_115	Hospital_5	F	85	431	1	1	2	1
Chart_116	Hospital_6	M	67	431	1	1	2	1
Chart_117	Hospital_6	M	83	431	1	1	2	1
Chart_118	Hospital_6	F	78	431	1	1	2	1
Chart_119	Hospital_6	M	93	431	1	1	2	1
Chart_120	Hospital_6	F	87	431	1	1	2	1
Chart_121	Hospital_6	F	83	431	1	1	2	1
Chart_122	Hospital_7	F	76	431	1	1	2	1
Chart_123	Hospital_7	M	88	431	1	1	2	1
Chart_124	Hospital_7	F	74	431	1	0	2	0
Chart_125	Hospital_7	F	87	431	1	1	2	1
Chart_126	Hospital_7	F	92	431	1	1	2	1
Chart_127	Hospital_7	M	83	431	1	1	2	1
Chart_128	Hospital_7	M	59	431	1	1	2	1
Chart_129	Hospital_7	M	95	431	1	1	2	1
Chart_130	Hospital_7	M	86	431	1	1	2	1

Legend: 0=no; 1=yes; 2=not reported; n.a.=clinical chart not available

Other and unspecified intracranial hemorrhage

Number Clinical Chart	Hospital	Gender	Patient's age on admission	Primary Diagnosis (ICD-9)	Presence of neurological signs, symptoms or coma	Positive CT or MRI	Other positive instrumental test	Validation
Chart_1	Hospital_1	M	36	4321	1	1	0	1
Chart_2	Hospital_1	M	62	4321	1	1	2	1
Chart_3	Hospital_1	F	64	4321	1	1	2	1
Chart_4	Hospital_1	M	72	4321	1	1	2	1
Chart_5	Hospital_1	M	75	4321	1	1	2	1
Chart_6	Hospital_1	M	81	4321	1	1	2	1
Chart_7	Hospital_1	M	83	4321	1	1	2	1
Chart_8	Hospital_1	M	76	4321	0	1	0	0
Chart_9	Hospital_1	M	65	4321	1	1	2	1
Chart_10	Hospital_1	F	90	4321	1	1	2	1
Chart_11	Hospital_1	M	91	4321	1	0	2	0
Chart_12	Hospital_1	M	73	4321	1	1	2	1
Chart_13	Hospital_1	F	77	4321	1	1	2	1
Chart_14	Hospital_1	F	74	4321	1	1	2	1
Chart_15	Hospital_1	M	75	4321	1	1	2	1
Chart_16	Hospital_1	F	49	4321	1	1	0	1
Chart_17	Hospital_1	M	87	4321	1	1	2	1
Chart_18	Hospital_1	M	71	4321	1	1	2	1
Chart_19	Hospital_1	M	89	4321	1	1	2	1
Chart_20	Hospital_1	M	83	4321	1	1	2	1
Chart_21	Hospital_1	M	78	4320	1	1	2	1
Chart_22	Hospital_1	F	70	4329	1	1	2	1
Chart_23	Hospital_1	F	80	4321	1	1	2	1
Chart_24	Hospital_1	F	77	4321	1	1	2	1
Chart_25	Hospital_1	M	84	4321	1	1	2	1
Chart_26	Hospital_1	F	81	4321	1	1	2	1
Chart_27	Hospital_1	M	89	4321	1	1	2	1
Chart_28	Hospital_1	M	90	4321	1	1	2	1
Chart_29	Hospital_1	F	77	4321	1	1	2	1
Chart_30	Hospital_1	F	69	4321	1	1	2	1
Chart_31	Hospital_1	M	83	4321	1	1	2	1
Chart_32	Hospital_1	M	79	4321	1	1	2	1

Number Clinical Chart	Hospital	Gender	Patient's age on admission	Primary Diagnosis (ICD-9)	Presence of neurological signs, symptoms or coma	Positive CT or MRI	Other positive instrumental test	Validation
Chart_33	Hospital_1	F	80	4329	1	1	2	1
Chart_34	Hospital_1	M	38	4321	1	1	2	1
Chart_35	Hospital_1	M	91	4320	1	1	2	1
Chart_36	Hospital_1	M	89	4321	1	1	2	1
Chart_37	Hospital_1	F	38	4321	1	1	2	1
Chart_38	Hospital_1	F	91	4321	1	1	2	1
Chart_39	Hospital_1	M	80	4321	1	1	2	1
Chart_40	Hospital_1	M	60	4321	1	1	2	1
Chart_41	Hospital_1	M	95	4321	1	1	2	1
Chart_42	Hospital_2	M	76	4329	1	1	2	1
Chart_43	Hospital_2	F	90	4321	1	1	2	1
Chart_44	Hospital_2	M	88	4321	1	1	2	1
Chart_45	Hospital_2	F	82	4321	1	1	2	1
Chart_46	Hospital_2	M	86	4321	1	1	2	1
Chart_47	Hospital_2	F	83	4321	1	1	2	1
Chart_48	Hospital_2	F	84	4321	1	1	2	1
Chart_49	Hospital_2	F	67	4321	1	1	2	1
Chart_50	Hospital_2	M	88	4321	1	1	2	1
Chart_51	Hospital_2	F	68	4321	1	1	2	1
Chart_52	Hospital_2	F	73	4321	1	1	2	1
Chart_53	Hospital_2	M	75	4321	1	1	2	1
Chart_54	Hospital_2	M	77	4321	1	1	2	1
Chart_55	Hospital_2	F	72	4321	1	1	2	1
Chart_56	Hospital_2	M	67	4321	1	1	2	1
Chart_57	Hospital_2	M	69	4321	1	1	2	1
Chart_58	Hospital_2	F	70	4321	1	1	2	1
Chart_59	Hospital_2	M	38	4321	1	1	2	1
Chart_60	Hospital_2	F	76	4321	1	1	2	1
Chart_61	Hospital_2	F	74	4321	1	1	2	1
Chart_62	Hospital_2	F	85	4321	1	1	2	1
Chart_63	Hospital_2	M	65	4321	1	1	2	1
Chart_64	Hospital_2	F	79	4321	1	1	0	1
Chart_65	Hospital_2	M	31	4321	1	1	2	1
Chart_66	Hospital_2	M	88	4321	1	1	2	1

Number Clinical Chart	Hospital	Gender	Patient's age on admission	Primary Diagnosis (ICD-9)	Presence of neurological signs, symptoms or coma	Positive CT or MRI	Other positive instrumental test	Validation
Chart_67	Hospital_2	M	54	4321	1	1	2	1
Chart_68	Hospital_2	M	87	4321	1	1	2	1
Chart_69	Hospital_2	F	89	4321	1	1	2	1
Chart_70	Hospital_2	M	85	4321	1	1	2	1
Chart_71	Hospital_2	M	72	4321	1	1	2	1
Chart_72	Hospital_2	M	80	4321	1	1	2	1
Chart_73	Hospital_2	M	87	4321	1	1	2	1
Chart_74	Hospital_2	M	67	4321	1	1	2	1
Chart_75	Hospital_2	M	66	4321	1	1	2	1
Chart_76	Hospital_2	F	69	4321	1	1	2	1
Chart_77	Hospital_2	F	67	4321	1	0	0	0
Chart_78	Hospital_2	M	77	4321	1	1	2	1
Chart_79	Hospital_2	M	87	4321	1	1	2	1
Chart_80	Hospital_2	M	46	4321	1	1	2	1
Chart_81	Hospital_2	M	75	4321	1	1	2	1
Chart_82	Hospital_2	M	97	4321	1	1	2	1
Chart_83	Hospital_2	M	88	4321	1	1	2	1
Chart_84	Hospital_2	F	52	4321	1	1	2	1
Chart_85	Hospital_2	M	46	4321	1	1	0	1
Chart_86	Hospital_2	F	87	4321	1	1	2	1
Chart_87	Hospital_2	M	70	4321	1	1	2	1
Chart_88	Hospital_2	F	--	4321	n.a.	n.a.	n.a.	n.a.
Chart_89	Hospital_2	F	81	4321	1	1	2	1
Chart_90	Hospital_2	M	72	4321	1	1	2	1
Chart_91	Hospital_2	F	80	4321	1	1	2	1
Chart_92	Hospital_2	M	86	4321	1	1	2	1
Chart_93	Hospital_2	M	63	4321	1	1	2	1
Chart_94	Hospital_2	F	77	4321	1	1	2	1
Chart_95	Hospital_2	M	78	4321	1	1	2	1
Chart_96	Hospital_2	F	73	4321	1	1	2	1
Chart_97	Hospital_2	M	75	4321	1	1	2	1
Chart_98	Hospital_2	M	77	4321	1	1	2	1
Chart_99	Hospital_2	F	96	4321	1	1	2	1
Chart_100	Hospital_2	F	77	4321	1	1	2	1

Number Clinical Chart	Hospital	Gender	Patient's age on admission	Primary Diagnosis (ICD-9)	Presence of neurological signs, symptoms or coma	Positive CT or MRI	Other positive instrumental test	Validation
Chart_101	Hospital_2	F	71	4321	1	1	2	1
Chart_102	Hospital_2	M	87	4321	1	1	1	1
Chart_103	Hospital_2	F	81	4321	1	1	2	1
Chart_104	Hospital_2	F	88	4321	1	1	2	1
Chart_105	Hospital_2	F	71	4321	1	1	2	1
Chart_106	Hospital_2	F	85	4321	1	1	2	1
Chart_107	Hospital_2	M	87	4321	1	1	2	1
Chart_108	Hospital_2	F	57	4321	1	1	2	1
Chart_109	Hospital_2	M	77	4321	1	1	2	1
Chart_110	Hospital_3	M	88	4321	1	1	2	1
Chart_111	Hospital_3	M	44	4320	1	1	2	1
Chart_112	Hospital_3	M	78	4321	1	1	2	1
Chart_113	Hospital_4	F	95	4321	1	1	2	1
Chart_114	Hospital_4	F	86	4321	1	1	2	1
Chart_115	Hospital_4	F	94	4321	1	1	2	1
Chart_116	Hospital_4	M	77	4321	1	1	2	1
Chart_117	Hospital_5	M	84	4321	1	1	2	1
Chart_118	Hospital_5	F	85	4329	1	1	2	1
Chart_119	Hospital_5	F	93	4321	1	1	2	1
Chart_120	Hospital_5	M	91	4321	1	1	2	1
Chart_121	Hospital_5	F	71	4321	1	1	2	1
Chart_122	Hospital_6	F	99	4321	1	1	2	1
Chart_123	Hospital_6	M	87	4321	1	1	2	1
Chart_124	Hospital_6	M	86	4321	1	1	2	1
Chart_125	Hospital_6	M	80	4320	1	1	2	1
Chart_126	Hospital_6	M	85	4321	1	1	2	1
Chart_127	Hospital_7	F	80	4321	1	1	2	1
Chart_128	Spoleto	M	86	4321	1	1	2	1
Chart_129	Spoleto	M	83	4321	1	1	2	1
Chart_130	Spoleto	M	84	4321	1	1	2	1

Legend: 0=no; 1=yes; 2=not reported; n.a.=clinical chart not available

Occlusion and stenosis of paracerebral arteries

Number Clinical Chart	Hospital	Gender	Patient's age on admission	Primary Diagnosis (ICD-9)	Presence of neurological signs, symptoms or coma	Positive CT or MRI	Other positive instrumental test	Validation
Chart_1	Hospital_1	M	85	43311	1	1	1	1
Chart_2	Hospital_1	F	79	43331	1	1	1	1
Chart_3	Hospital_1	F	67	43331	0	2	1	0
Chart_4	Hospital_1	M	80	43311	0	2	1	0
Chart_5	Hospital_1	F	64	43311	0	2	1	0
Chart_6	Hospital_1	M	80	43311	1	1	1	1
Chart_7	Hospital_1	M	64	43311	0	2	1	0
Chart_8	Hospital_1	F	71	43311	0	2	1	0
Chart_9	Hospital_1	F	76	43311	1	1	1	1
Chart_10	Hospital_1	F	80	43311	1	1	2	1
Chart_11	Hospital_1	M	77	43311	0	2	1	0
Chart_12	Hospital_1	F	69	43311	0	2	1	0
Chart_13	Hospital_1	F	87	43301	1	1	2	1
Chart_14	Hospital_1	F	83	43311	1	1	1	1
Chart_15	Hospital_1	M	75	43311	0	2	1	0
Chart_16	Hospital_1	F	87	43311	1	0	1	1
Chart_17	Hospital_1	M	75	43311	1	1	1	1
Chart_18	Hospital_1	F	66	43311	1	1	1	1
Chart_19	Hospital_1	M	76	43311	0	2	1	0
Chart_20	Hospital_1	F	64	43311	0	0	1	0
Chart_21	Hospital_1	F	59	43311	1	1	1	1
Chart_22	Hospital_1	F	70	43311	0	2	1	0
Chart_23	Hospital_1	F	77	43311	1	0	1	1
Chart_24	Hospital_1	M	56	43311	0	2	1	0
Chart_25	Hospital_1	M	76	43311	0	2	1	0
Chart_26	Hospital_1	M	89	43311	1	1	1	1
Chart_27	Hospital_1	F	92	43311	1	1	1	1
Chart_28	Hospital_1	M	65	43311	1	1	2	0
Chart_29	Hospital_1	M	81	43311	1	0	1	1
Chart_30	Hospital_1	M	63	43311	1	1	1	1
Chart_31	Hospital_1	F	69	43311	1	1	0	1
Chart_32	Hospital_1	M	68	43311	0	2	1	0

Number Clinical Chart	Hospital	Gender	Patient's age on admission	Primary Diagnosis (ICD-9)	Presence of neurological signs, symptoms or coma	Positive CT or MRI	Other positive instrumental test	Validation
Chart_33	Hospital_1	M	76	43301	1	1	2	1
Chart_34	Hospital_1	M	68	43311	0	0	1	0
Chart_35	Hospital_1	M	73	43311	1	1	1	1
Chart_36	Hospital_1	M	63	43311	1	1	1	1
Chart_37	Hospital_1	M	83	43301	1	1	1	1
Chart_38	Hospital_1	F	79	43311	1	2	1	1
Chart_39	Hospital_1	F	78	43311	0	2	1	0
Chart_40	Hospital_1	M	77	43311	0	2	1	0
Chart_41	Hospital_1	F	65	43311	0	2	1	0
Chart_42	Hospital_1	F	82	43311	1	1	2	0
Chart_43	Hospital_1	F	69	43311	1	1	1	1
Chart_44	Hospital_1	F	78	43311	1	1	1	1
Chart_45	Hospital_1	F	81	43311	1	1	2	0
Chart_46	Hospital_1	M	79	43311	1	1	1	1
Chart_47	Hospital_1	M	78	43311	1	0	1	1
Chart_48	Hospital_1	F	87	43311	1	1	1	1
Chart_49	Hospital_1	M	71	43311	2	2	1	0
Chart_50	Hospital_1	M	77	43311	1	1	1	1
Chart_51	Hospital_1	F	89	43311	1	1	1	1
Chart_52	Hospital_1	F	75	43311	1	2	1	1
Chart_53	Hospital_1	F	75	43311	1	0	1	1
Chart_54	Hospital_1	M	73	43311	1	0	2	0
Chart_55	Hospital_1	M	--	43311	n.a.	n.a.	n.a.	n.a.
Chart_56	Hospital_1	F	68	43311	1	1	1	1
Chart_57	Hospital_1	M	82	43311	1	0	1	1
Chart_58	Hospital_1	F	74	43311	2	0	1	0
Chart_59	Hospital_1	F	80	43311	1	0	0	0
Chart_60	Hospital_1	M	62	43311	2	2	1	0
Chart_61	Hospital_1	M	77	43311	2	2	1	0
Chart_62	Hospital_1	M	74	43311	2	2	1	0
Chart_63	Hospital_1	F	76	43311	2	2	1	0
Chart_64	Hospital_1	M	79	43311	1	1	1	1
Chart_65	Hospital_1	M	70	43331	1	1	1	1
Chart_66	Hospital_1	F	69	43311	2	2	1	0

Number Clinical Chart	Hospital	Gender	Patient's age on admission	Primary Diagnosis (ICD-9)	Presence of neurological signs, symptoms or coma	Positive CT or MRI	Other positive instrumental test	Validation
Chart_67	Hospital_1	F	92	43311	1	1	1	1
Chart_68	Hospital_1	M	58	43311	1	1	1	1
Chart_69	Hospital_1	M	87	43311	1	1	1	1
Chart_70	Hospital_1	F	69	43311	1	1	1	1
Chart_71	Hospital_1	M	69	43311	1	1	1	1
Chart_72	Hospital_1	M	77	43311	1	1	1	1
Chart_73	Hospital_1	M	62	43311	1	1	1	1
Chart_74	Hospital_1	M	67	43311	1	1	1	1
Chart_75	Hospital_1	M	67	43311	1	1	1	1
Chart_76	Hospital_1	M	79	43311	1	2	1	1
Chart_77	Hospital_2	M	79	43311	1	1	1	1
Chart_78	Hospital_2	M	62	43311	1	0	2	0
Chart_79	Hospital_2	M	49	43321	1	1	2	1
Chart_80	Hospital_2	F	88	43311	1	1	1	1
Chart_81	Hospital_2	M	78	43311	1	1	1	1
Chart_82	Hospital_2	M	71	43321	1	1	1	1
Chart_83	Hospital_2	M	71	43311	1	2	1	1
Chart_84	Hospital_2	M	75	43311	1	1	1	1
Chart_85	Hospital_2	M	74	43311	1	2	1	1
Chart_86	Hospital_2	M	75	43331	1	1	2	1
Chart_87	Hospital_2	F	83	43311	1	1	1	1
Chart_88	Hospital_2	M	94	43311	1	0	0	0
Chart_89	Hospital_2	F	77	43311	1	1	1	1
Chart_90	Hospital_2	M	89	43311	2	2	1	0
Chart_91	Hospital_2	M	51	43311	1	1	1	1
Chart_92	Hospital_2	M	76	43311	1	1	1	1
Chart_93	Hospital_2	F	69	43311	1	1	1	1
Chart_94	Hospital_2	M	61	43311	1	1	1	1
Chart_95	Hospital_2	M	77	43311	1	2	1	1
Chart_96	Hospital_2	M	75	43311	1	0	1	1
Chart_97	Hospital_2	M	88	43311	1	0	2	0
Chart_98	Hospital_2	M	61	43311	1	1	1	1
Chart_99	Hospital_2	M	70	43311	1	1	1	1
Chart_100	Hospital_2	M	39	43311	1	1	1	1

Number Clinical Chart	Hospital	Gender	Patient's age on admission	Primary Diagnosis (ICD-9)	Presence of neurological signs, symptoms or coma	Positive CT or MRI	Other positive instrumental test	Validation
Chart_101	Hospital_2	F	75	43311	1	2	1	1
Chart_102	Hospital_2	M	74	43311	1	1	1	1
Chart_103	Hospital_2	M	83	43311	1	1	1	1
Chart_104	Hospital_2	M	--	43321	n.a.	n.a.	n.a.	n.a.
Chart_105	Hospital_2	M	81	43311	1	1	1	1
Chart_106	Hospital_3	F	84	43311	1	0	1	1
Chart_107	Hospital_3	M	67	43311	1	1	1	1
Chart_108	Hospital_3	M	87	43311	1	1	1	1
Chart_109	Hospital_3	F	65	43311	1	1	1	1
Chart_110	Hospital_3	F	93	43311	1	1	1	1
Chart_111	Hospital_3	M	82	43311	2	0	1	0
Chart_112	Hospital_3	M	88	43311	1	1	1	1
Chart_113	Hospital_5	M	79	43311	1	0	2	0
Chart_114	Hospital_5	M	63	43311	2	2	1	0
Chart_115	Hospital_5	M	89	43311	1	1	2	1
Chart_116	Hospital_5	M	80	43311	2	2	1	0
Chart_117	Hospital_5	M	73	43311	1	1	1	1
Chart_118	Hospital_5	M	79	43311	1	1	1	1
Chart_119	Hospital_5	M	76	43311	1	0	1	1
Chart_120	Hospital_5	M	82	43311	1	0	2	0
Chart_121	Hospital_5	M	68	43311	1	2	1	1
Chart_122	Hospital_5	M	77	43311	1	0	1	1
Chart_123	Hospital_5	M	83	43311	1	0	1	1
Chart_124	Hospital_6	M	76	43311	1	1	1	1
Chart_125	Hospital_6	M	82	43311	1	0	1	1
Chart_126	Hospital_6	M	87	43311	1	0	1	1
Chart_127	Hospital_6	M	66	43311	1	1	1	1
Chart_128	Hospital_6	M	86	43311	1	1	1	1
Chart_129	Hospital_6	F	77	43311	1	0	1	1
Chart_130	Hospital_6	F	96	43311	1	1	1	1

Legend: 0=no; 1=yes; 2=not reported; n.a.=clinical chart not available

Occlusion of cerebral arteries

Number Clinical Chart	Hospital	Gender	Patient's age on admission	Primary Diagnosis (ICD-9)	Presence of neurological signs, symptoms or coma	Positive CT or MRI	Other positive instrumental test	Validation
Chart_1	Hospital_1	M	86	43411	1	1	1	1
Chart_2	Hospital_1	F	61	43491	1	1	0	1
Chart_3	Hospital_1	F	95	43491	1	1	1	1
Chart_4	Hospital_1	M	81	43491	1	1	2	1
Chart_5	Hospital_1	M	61	43411	1	1	2	1
Chart_6	Hospital_1	F	78	43401	1	1	2	1
Chart_7	Hospital_1	F	24	43401	1	1	1	1
Chart_8	Hospital_1	F	92	43491	1	1	2	1
Chart_9	Hospital_1	F	71	43401	1	1	2	1
Chart_10	Hospital_1	F	89	43411	1	1	2	1
Chart_11	Hospital_1	M	93	43411	1	1	1	1
Chart_12	Hospital_1	F	92	43401	1	1	2	1
Chart_13	Hospital_1	M	86	43401	1	1	2	1
Chart_14	Hospital_1	M	58	43401	1	1	2	1
Chart_15	Hospital_1	M	49	43411	1	1	2	1
Chart_16	Hospital_1	F	67	43401	1	1	2	1
Chart_17	Hospital_1	M	63	43401	1	1	2	1
Chart_18	Hospital_1	M	77	43401	1	1	2	1
Chart_19	Hospital_1	M	53	43491	1	1	2	1
Chart_20	Hospital_1	M	91	43401	1	1	2	1
Chart_21	Hospital_1	M	80	43411	1	1	2	1
Chart_22	Hospital_1	M	88	43411	1	1	2	1
Chart_23	Hospital_1	M	70	43401	1	1	2	1
Chart_24	Hospital_1	F	79	43401	1	1	2	1
Chart_25	Hospital_1	M	73	43401	1	1	2	1
Chart_26	Hospital_1	M	91	43401	1	1	0	1
Chart_27	Hospital_1	F	77	43401	1	1	2	1
Chart_28	Hospital_1	F	89	43401	1	1	2	1
Chart_29	Hospital_1	F	--	43401	n.a.	n.a.	n.a.	n.a.
Chart_30	Hospital_1	M	71	43411	1	1	2	1
Chart_31	Hospital_1	M	58	43401	1	1	2	1
Chart_32	Hospital_1	M	74	43491	1	1	2	1

Number Clinical Chart	Hospital	Gender	Patient's age on admission	Primary Diagnosis (ICD-9)	Presence of neurological signs, symptoms or coma	Positive CT or MRI	Other positive instrumental test	Validation
Chart_33	Hospital_1	F	80	43411	1	1	1	1
Chart_34	Hospital_1	M	68	43411	1	1	2	1
Chart_35	Hospital_1	M	79	43411	1	1	2	1
Chart_36	Hospital_1	F	83	43401	1	1	2	1
Chart_37	Hospital_1	M	40	43401	1	1	2	1
Chart_38	Hospital_1	M	57	43411	1	1	1	1
Chart_39	Hospital_1	M	58	43401	1	1	2	1
Chart_40	Hospital_1	M	84	43411	1	1	2	1
Chart_41	Hospital_1	F	85	43401	0	0	0	0
Chart_42	Hospital_1	F	51	43401	1	1	2	1
Chart_43	Hospital_1	F	90	43401	1	1	2	1
Chart_44	Hospital_1	F	89	43401	1	1	2	1
Chart_45	Hospital_1	M	74	43401	1	1	2	1
Chart_46	Hospital_1	M	65	43401	1	1	2	1
Chart_47	Hospital_2	F	81	43411	1	1	2	1
Chart_48	Hospital_2	M	66	43401	1	0	2	0
Chart_49	Hospital_2	M	76	43401	1	0	0	0
Chart_50	Hospital_2	M	77	43401	1	1	2	1
Chart_51	Hospital_2	F	80	43401	1	1	2	1
Chart_52	Hospital_2	M	66	43401	1	1	2	1
Chart_53	Hospital_2	F	83	43401	1	0	0	0
Chart_54	Hospital_2	M	62	43401	1	1	2	1
Chart_55	Hospital_2	M	47	43401	1	1	2	1
Chart_56	Hospital_2	M	77	43401	1	1	2	1
Chart_57	Hospital_2	F	67	43401	1	1	2	1
Chart_58	Hospital_2	M	43	43401	1	1	2	1
Chart_59	Hospital_2	M	56	43401	1	1	2	1
Chart_60	Hospital_2	M	65	43401	1	1	2	1
Chart_61	Hospital_2	M	79	43401	1	1	2	1
Chart_62	Hospital_2	M	84	43401	1	1	2	1
Chart_63	Hospital_2	M	90	43401	1	2	1	0
Chart_64	Hospital_2	F	47	43401	1	1	2	1
Chart_65	Hospital_2	F	79	43401	1	1	2	1
Chart_66	Hospital_2	F	76	43401	1	1	2	1

Number Clinical Chart	Hospital	Gender	Patient's age on admission	Primary Diagnosis (ICD-9)	Presence of neurological signs, symptoms or coma	Positive CT or MRI	Other positive instrumental test	Validation
Chart_67	Hospital_2	M	87	43401	1	1	2	1
Chart_68	Hospital_2	F	77	43401	1	1	2	1
Chart_69	Hospital_2	F	73	43411	1	1	2	1
Chart_70	Hospital_2	F	89	43401	1	1	2	1
Chart_71	Hospital_3	M	71	43401	1	1	2	1
Chart_72	Hospital_3	M	72	43401	1	1	2	1
Chart_73	Hospital_3	F	82	43411	1	1	2	1
Chart_74	Hospital_3	F	87	43411	1	0	1	0
Chart_75	Hospital_3	F	77	43401	1	1	2	1
Chart_76	Hospital_3	F	87	43401	1	1	2	1
Chart_77	Hospital_3	M	85	43401	1	1	2	1
Chart_78	Hospital_3	M	66	43401	1	1	2	1
Chart_79	Hospital_3	M	68	43401	1	1	1	1
Chart_80	Hospital_3	M	58	43401	1	1	2	1
Chart_81	Hospital_3	F	93	43411	1	0	2	0
Chart_82	Hospital_3	M	90	43491	1	1	2	1
Chart_83	Hospital_3	F	82	43491	1	1	2	1
Chart_84	Hospital_3	F	85	43491	1	1	2	1
Chart_85	Hospital_3	M	69	43491	1	1	2	1
Chart_86	Hospital_3	M	79	43401	1	1	2	1
Chart_87	Hospital_3	F	77	43491	1	1	2	1
Chart_88	Hospital_3	M	48	43491	1	1	2	1
Chart_89	Hospital_3	M	92	43491	1	0	1	0
Chart_90	Hospital_3	M	80	43401	1	1	2	1
Chart_91	Hospital_3	F	84	43491	1	0	2	0
Chart_92	Hospital_3	F	93	43491	1	0	2	0
Chart_93	Hospital_4	F	64	43491	1	1	2	1
Chart_94	Hospital_4	M	78	43401	1	0	0	0
Chart_95	Hospital_4	M	90	43401	1	1	2	1
Chart_96	Hospital_4	M	95	43411	1	1	2	1
Chart_97	Hospital_4	F	87	43401	1	1	2	1
Chart_98	Hospital_4	M	84	43411	1	1	2	1
Chart_99	Hospital_4	F	89	43401	1	1	2	1
Chart_100	Hospital_4	F	93	43411	1	1	2	1

Number Clinical Chart	Hospital	Gender	Patient's age on admission	Primary Diagnosis (ICD-9)	Presence of neurological signs, symptoms or coma	Positive CT or MRI	Other positive instrumental test	Validation
Chart_101	Hospital_4	F	69	43401	1	1	2	1
Chart_102	Hospital_4	M	90	43401	1	1	2	1
Chart_103	Hospital_4	F	77	43401	1	1	2	1
Chart_104	Hospital_4	F	79	43411	1	1	2	1
Chart_105	Hospital_4	M	71	43401	1	1	2	1
Chart_106	Hospital_5	M	71	43401	1	1	2	1
Chart_107	Hospital_5	F	53	43491	1	1	2	1
Chart_108	Hospital_5	F	64	43401	1	0	2	0
Chart_109	Hospital_5	F	89	43401	1	1	2	1
Chart_110	Hospital_5	F	81	43401	1	1	2	1
Chart_111	Hospital_5	F	82	43401	1	1	2	1
Chart_112	Hospital_5	M	63	43401	1	1	2	1
Chart_113	Hospital_5	F	78	43401	1	1	2	1
Chart_114	Hospital_5	M	81	43401	1	1	2	1
Chart_115	Hospital_5	M	67	43401	1	1	2	1
Chart_116	Hospital_5	M	48	43401	1	1	2	1
Chart_117	Hospital_5	M	88	43411	1	1	2	1
Chart_118	Hospital_5	F	78	43401	1	1	2	1
Chart_119	Hospital_5	M	90	43401	1	1	2	1
Chart_120	Hospital_6	F	71	43401	1	1	2	1
Chart_121	Hospital_6	M	72	43491	1	1	2	1
Chart_122	Hospital_6	F	91	43401	1	1	2	1
Chart_123	Hospital_6	F	73	43401	1	1	2	1
Chart_124	Hospital_6	F	75	43401	1	1	2	1
Chart_125	Hospital_6	F	79	43401	1	1	1	1
Chart_126	Hospital_6	M	50	43401	1	1	2	1
Chart_127	Hospital_6	F	88	43401	1	1	2	1
Chart_128	Hospital_6	F	84	43411	1	1	2	1
Chart_129	Hospital_6	F	90	43401	1	1	2	1
Chart_130	Hospital_7	M	91	43401	1	1	2	1

Legend: 0=no; 1=yes; 2=not reported; n.a.=clinical chart not available

Acute but ill-defined cerebrovascular disease

Number Clinical Chart	Hospital	Gender	Patient's age on admission	Primary Diagnosis (ICD-9)	Presence of neurological signs, symptoms or coma	Positive CT or MRI	Other positive instrumental test	Validation
Chart_1	Hospital_1	M	84	436	1	1	2	1
Chart_2	Hospital_1	F	82	436	1	1	2	1
Chart_3	Hospital_1	F	78	436	1	1	2	1
Chart_4	Hospital_1	F	90	436	1	1	2	1
Chart_5	Hospital_1	M	74	436	1	1	2	1
Chart_6	Hospital_1	M	76	436	1	1	2	1
Chart_7	Hospital_1	M	62	436	1	1	2	1
Chart_8	Hospital_1	F	77	436	1	1	2	1
Chart_9	Hospital_1	F	94	436	1	0	2	0
Chart_10	Hospital_1	M	83	436	1	1	2	1
Chart_11	Hospital_1	M	76	436	1	1	2	1
Chart_12	Hospital_1	F	88	436	1	1	2	1
Chart_13	Hospital_1	F	89	436	1	2	2	0
Chart_14	Hospital_1	M	81	436	n.a.	n.a.	n.a.	n.a.
Chart_15	Hospital_1	F	81	436	1	1	2	1
Chart_16	Hospital_1	M	71	436	1	1	2	1
Chart_17	Hospital_1	F	72	436	1	1	2	1
Chart_18	Hospital_1	F	90	436	0	1	2	0
Chart_19	Hospital_1	F	88	436	1	1	2	1
Chart_20	Hospital_1	M	70	436	1	1	2	1
Chart_21	Hospital_1	F	81	436	1	1	2	1
Chart_22	Hospital_1	F	85	436	1	1	2	1
Chart_23	Hospital_1	M	92	436	1	1	2	1
Chart_24	Hospital_1	F	69	436	1	1	2	1
Chart_25	Hospital_1	F	52	436	1	1	2	1
Chart_26	Hospital_1	F	75	436	1	1	2	1
Chart_27	Hospital_1	M	73	436	1	0	1	1
Chart_28	Hospital_1	F	72	436	1	0	1	0
Chart_29	Hospital_1	F	76	436	1	1	2	1
Chart_30	Hospital_1	M	83	436	1	1	2	1
Chart_31	Hospital_1	F	69	436	1	1	2	1
Chart_32	Hospital_1	F	90	436	1	1	2	1
Chart_33	Hospital_1	M	83	436	1	1	2	1

Number Clinical Chart	Hospital	Gender	Patient's age on admission	Primary Diagnosis (ICD-9)	Presence of neurological signs, symptoms or coma	Positive CT or MRI	Other positive instrumental test	Validation
Chart_34	Hospital_1	F	65	436	1	1	2	1
Chart_35	Hospital_1	M	73	436	1	1	2	1
Chart_36	Hospital_1	F	84	436	1	1	2	1
Chart_37	Hospital_1	M	81	436	1	1	2	1
Chart_38	Hospital_1	M	78	436	1	1	2	1
Chart_39	Hospital_1	M	67	436	1	1	2	1
Chart_40	Hospital_1	M	77	436	1	1	2	1
Chart_41	Hospital_1	M	76	436	1	1	2	1
Chart_42	Hospital_1	F	73	436	1	1	2	1
Chart_43	Hospital_1	M	78	436	1	1	2	1
Chart_44	Hospital_1	M	77	436	1	1	2	1
Chart_45	Hospital_1	F	95	436	1	1	2	1
Chart_46	Hospital_1	F	85	436	1	1	2	1
Chart_47	Hospital_1	M	93	436	1	1	2	1
Chart_48	Hospital_1	F	86	436	1	1	2	1
Chart_49	Hospital_1	F	63	436	1	1	2	1
Chart_50	Hospital_1	F	97	436	1	1	2	1
Chart_51	Hospital_1	F	93	436	1	1	2	1
Chart_52	Hospital_1	F	86	436	1	1	2	1
Chart_53	Hospital_1	F	93	436	1	1	2	1
Chart_54	Hospital_1	F	--	436	n.a.	n.a.	n.a.	n.a.
Chart_55	Hospital_1	M	90	436	1	1	2	1
Chart_56	Hospital_1	F	74	436	1	1	2	1
Chart_57	Hospital_1	M	85	436	1	1	2	1
Chart_58	Hospital_1	M	90	436	1	1	2	1
Chart_59	Hospital_1	M	59	436	1	1	2	1
Chart_60	Hospital_1	F	80	436	1	0	2	0
Chart_61	Hospital_1	F	87	436	0	0	2	0
Chart_62	Hospital_2	F	88	436	1	2	2	0
Chart_63	Hospital_2	F	95	436	1	1	2	1
Chart_64	Hospital_2	M	88	436	1	0	2	0
Chart_65	Hospital_2	M	87	436	1	1	2	1
Chart_66	Hospital_2	M	89	436	1	1	2	1
Chart_67	Hospital_2	F	82	436	1	1	2	1
Chart_68	Hospital_2	F	96	436	1	1	2	1

Number Clinical Chart	Hospital	Gender	Patient's age on admission	Primary Diagnosis (ICD-9)	Presence of neurological signs, symptoms or coma	Positive CT or MRI	Other positive instrumental test	Validation
Chart_69	Hospital_2	F	79	436	1	1	2	1
Chart_70	Hospital_2	M	90	436	1	1	2	1
Chart_71	Hospital_2	F	92	436	1	1	2	1
Chart_72	Hospital_2	M	81	436	1	1	2	1
Chart_73	Hospital_2	M	95	436	1	1	2	1
Chart_74	Hospital_2	M	72	436	1	1	2	1
Chart_75	Hospital_2	F	74	436	1	1	2	1
Chart_76	Hospital_2	F	95	436	1	1	2	1
Chart_77	Hospital_2	M	75	436	1	1	2	1
Chart_78	Hospital_2	F	59	436	1	1	2	1
Chart_79	Hospital_2	F	97	436	1	1	2	1
Chart_80	Hospital_2	F	88	436	1	1	2	1
Chart_81	Hospital_2	F	92	436	1	1	2	1
Chart_82	Hospital_2	F	57	436	1	0	2	0
Chart_83	Hospital_2	F	89	436	1	1	2	1
Chart_84	Hospital_2	M	63	436	1	1	2	1
Chart_85	Hospital_2	F	86	436	1	1	2	1
Chart_86	Hospital_2	F	96	436	0	1	2	0
Chart_87	Hospital_3	F	82	436	1	0	2	0
Chart_88	Hospital_3	F	67	436	1	2	2	0
Chart_89	Hospital_3	F	85	436	1	0	2	0
Chart_90	Hospital_3	F	95	436	1	2	2	0
Chart_91	Hospital_3	F	84	436	1	2	2	0
Chart_92	Hospital_3	F	82	436	1	1	2	1
Chart_93	Hospital_3	F	79	436	1	1	2	1
Chart_94	Hospital_3	M	72	436	1	1	2	1
Chart_95	Hospital_3	M	70	436	1	2	2	0
Chart_96	Hospital_3	F	78	436	1	2	2	0
Chart_97	Hospital_3	F	65	436	1	0	2	0
Chart_98	Hospital_3	M	86	436	1	1	2	1
Chart_99	Hospital_4	M	61	436	1	1	2	1
Chart_100	Hospital_4	F	80	436	1	1	2	1
Chart_101	Hospital_4	M	75	436	1	0	2	0
Chart_102	Hospital_4	M	81	436	1	1	2	1
Chart_103	Hospital_4	F	90	436	1	1	2	1

Number Clinical Chart	Hospital	Gender	Patient's age on admission	Primary Diagnosis (ICD-9)	Presence of neurological signs, symptoms or coma	Positive CT or MRI	Other positive instrumental test	Validation
Chart_104	Hospital_6	M	97	436	1	1	2	1
Chart_105	Hospital_6	F	86	436	1	1	2	1
Chart_106	Hospital_6	F	55	436	1	1	2	1
Chart_107	Hospital_6	F	84	436	1	1	2	1
Chart_108	Hospital_6	F	85	436	1	1	2	1
Chart_109	Hospital_6	F	80	436	1	1	2	1
Chart_110	Hospital_6	F	82	436	1	1	2	1
Chart_111	Hospital_6	M	62	436	1	1	2	1
Chart_112	Hospital_6	F	87	436	1	1	2	1
Chart_113	Hospital_6	M	84	436	1	1	2	1
Chart_114	Hospital_6	F	93	436	1	0	2	0
Chart_115	Hospital_6	M	83	436	1	1	2	1
Chart_116	Hospital_6	M	81	436	1	1	2	1
Chart_117	Hospital_6	F	90	436	1	1	2	1
Chart_118	Hospital_6	F	64	436	1	0	2	0
Chart_119	Hospital_6	M	89	436	1	1	2	1
Chart_120	Hospital_6	M	81	436	1	1	2	1
Chart_121	Hospital_6	F	89	436	1	1	2	1
Chart_122	Hospital_6	M	66	436	1	1	2	1
Chart_123	Hospital_6	M	76	436	1	1	2	1
Chart_124	Hospital_6	F	79	436	1	1	2	1
Chart_125	Hospital_6	F	88	436	1	1	2	1
Chart_126	Hospital_6	F	84	436	1	0	2	0
Chart_127	Hospital_6	M	73	436	1	1	2	1
Chart_128	Hospital_6	F	81	436	1	1	2	1
Chart_129	Hospital_6	F	91	436	1	1	2	1
Chart_130	Hospital_6	M	--	436	n.a.	n.a.	n.a.	n.a.

Legend: 0=no; 1=yes; 2=not reported; n.a.=clinical chart not available

Non-cases

Number Clinical Chart	Hospital	Gender	Patient's age at admission	Primary Diagnosis (ICD-9)	Non-cases group for 430 - Validation	Non-cases group for 431 - Validation	Non-cases group for 432.x - Validation	Non-cases group for 433.x1 - Validation	Non-cases group for 434.x1 - Validation	Non-cases group for 436 - Validation
Chart_1	Hospital_1	M	59	41519	1	1	1	0	1	1
Chart_2	Hospital_1	M	75	43330	1	1	1	1	1	1
Chart_3	Hospital_1	M	88	44421	1	1	1	1	1	1
Chart_4	Hospital_1	M	31	4270	1	1	1	1	1	1
Chart_5	Hospital_1	M	89	42654	1	1	1	1	1	1
Chart_6	Hospital_1	M	81	44021	1	1	1	1	1	1
Chart_7	Hospital_1	M	81	4281	1	1	1	1	1	1
Chart_8	Hospital_1	F	78	4139	1	1	1	1	1	1
Chart_9	Hospital_1	M	82	44024	1	1	1	1	1	1
Chart_10	Hospital_1	M	65	40211	1	1	1	1	1	1
Chart_11	Hospital_1	M	84	4370	1	1	1	1	1	1
Chart_12	Hospital_1	M	60	4359	1	1	1	1	1	1
Chart_13	Hospital_1	M	67	41071	1	1	1	1	1	1
Chart_14	Hospital_1	M	84	42781	1	1	1	1	1	1
Chart_15	Hospital_1	F	--	45111	n.a.	n.a.	n.a.	n.a.	n.a.	n.a.
Chart_16	Hospital_1	M	80	4299	1	1	1	1	1	1
Chart_17	Hospital_1	F	84	4379	1	1	1	1	1	1
Chart_18	Hospital_1	M	60	41071	1	1	1	1	1	1
Chart_19	Hospital_1	M	65	41071	1	1	1	1	1	1
Chart_20	Hospital_1	M	70	4148	1	1	1	1	1	1
Chart_21	Hospital_1	M	34	4564	1	1	1	1	1	1
Chart_22	Hospital_1	M	82	4414	1	1	1	1	1	1
Chart_23	Hospital_1	F	95	42731	1	1	1	1	1	1
Chart_24	Hospital_1	F	66	4373	1	1	1	1	1	1
Chart_25	Hospital_1	M	64	4270	1	1	1	1	1	1
Chart_26	Hospital_1	M	50	41051	1	1	1	1	1	1
Chart_27	Hospital_1	F	83	42781	1	1	1	1	1	1
Chart_28	Hospital_1	F	70	4358	1	1	1	1	1	1
Chart_29	Hospital_1	M	81	43320	1	1	1	1	1	1
Chart_30	Hospital_1	F	82	44422	1	1	1	1	1	1
Chart_31	Hospital_2	F	45	4538	1	1	1	1	1	1
Chart_32	Hospital_2	M	69	40290	1	1	1	1	1	1
Chart_33	Hospital_2	M	81	4359	1	1	1	1	1	1

Number Clinical Chart	Hospital	Gender	Patient's age at admission	Primary Diagnosis (ICD-9)	Non-cases group for 430 - Validation	Non-cases group for 431 - Validation	Non-cases group for 432.x - Validation	Non-cases group for 433.x1 - Validation	Non-cases group for 434.x1 - Validation	Non-cases group for 436 - Validation
Chart_34	Hospital_2	F	73	42731	1	1	1	1	1	1
Chart_35	Hospital_2	M	84	4414	1	1	1	1	1	1
Chart_36	Hospital_2	F	81	40211	1	1	1	1	1	1
Chart_37	Hospital_2	M	61	41401	1	1	1	1	1	1
Chart_38	Hospital_2	F	--	4280	n.a.	n.a.	n.a.	n.a.	n.a.	n.a.
Chart_39	Hospital_2	M	82	4280	1	1	1	1	1	1
Chart_40	Hospital_2	M	66	43310	1	1	1	0	1	1
Chart_41	Hospital_2	M	82	42731	1	1	1	1	1	1
Chart_42	Hospital_2	M	59	4011	1	1	1	1	1	1
Chart_43	Hospital_2	F	66	4541	1	1	1	1	1	1
Chart_44	Hospital_2	F	74	41071	1	1	1	1	1	1
Chart_45	Hospital_2	F	66	42731	1	1	1	1	1	1
Chart_46	Hospital_2	M	66	4414	1	1	1	1	1	1
Chart_47	Hospital_2	M	44	41401	1	1	1	1	1	1
Chart_48	Hospital_2	M	75	41401	1	1	1	1	1	1
Chart_49	Hospital_2	F	79	4254	1	1	1	1	1	1
Chart_50	Hospital_2	F	57	45119	1	1	1	1	1	1
Chart_51	Hospital_2	F	74	4019	1	1	1	1	1	1
Chart_52	Hospital_2	F	67	4240	1	1	1	1	1	1
Chart_53	Hospital_3	M	80	44022	1	1	1	1	1	1
Chart_54	Hospital_3	F	79	4374	1	1	1	1	1	1
Chart_55	Hospital_3	F	91	42843	1	1	1	1	1	1
Chart_56	Hospital_3	F	33	41011	1	1	1	1	1	1
Chart_57	Hospital_4	F	89	4260	1	1	1	1	1	1
Chart_58	Hospital_4	M	98	41071	1	1	1	1	1	1
Chart_59	Hospital_4	F	92	4281	1	1	1	1	1	1
Chart_60	Hospital_4	M	83	42781	1	1	1	1	1	1
Chart_61	Hospital_5	F	63	42983	1	1	1	1	1	1
Chart_62	Hospital_5	M	77	3950	1	1	1	1	1	1
Chart_63	Hospital_5	M	75	42731	1	1	1	1	1	1
Chart_64	Hospital_5	M	83	41071	1	1	1	1	1	1
Chart_65	Hospital_5	M	61	41041	1	1	1	1	1	1
Chart_66	Hospital_5	F	83	41071	1	1	1	1	1	1
Chart_67	Hospital_5	M	76	41011	1	1	1	1	1	1
Chart_68	Hospital_5	M	48	4241	1	1	1	1	1	1

Number Clinical Chart	Hospital	Gender	Patient's age at admission	Primary Diagnosis (ICD-9)	Non-cases group for 430 - Validation	Non-cases group for 431 - Validation	Non-cases group for 432.x - Validation	Non-cases group for 433.x1 - Validation	Non-cases group for 434.x1 - Validation	Non-cases group for 436 - Validation
Chart_69	Hospital_5	M	75	43889	1	1	1	1	1	1
Chart_70	Hospital_5	F	89	4280	1	1	1	1	1	1
Chart_71	Hospital_6	M	86	44024	1	1	1	1	1	1
Chart_72	Hospital_6	F	67	42761	1	1	1	1	1	1
Chart_73	Hospital_6	F	93	41071	1	1	1	1	1	1
Chart_74	Hospital_6	F	95	4168	1	1	1	1	1	1
Chart_75	Hospital_6	F	94	4358	1	1	1	1	1	1
Chart_76	Hospital_6	M	78	4139	1	1	1	1	1	1
Chart_77	Hospital_7	M	36	4555	1	1	1	1	1	1
Chart_78	Hospital_7	M	84	41071	1	1	1	1	1	1
Chart_79	Hospital_7	F	75	41071	1	1	1	1	1	1
Chart_80	Hospital_7	M	53	4359	1	1	1	1	1	1

Legend: 0=no; 1=yes; 2=not reported; n.a.=clinical chart not available
