# Supplementary material for: Validity of cerebrovascular ICD-9-CM codes in healthcare administrative databases. The Umbria Data-Value Project
Source: PLoS One. 2020 Jan 9;15(1):e0227653. doi: 10.1371/journal.pone.0227653 (PMC6952250; doi:10.1371/journal.pone.0227653)
Supplement: Attachment — Submitted filename: Response to Reviewers.docx [file pone.0227653.s003.docx]

**Response to Reviewers**

Da: PLOS ONE <em@editorialmanager.com>

A: Iosief Abraha <iosief_a@yahoo.it>

Inviato: mercoledì 6 novembre 2019, 10:59:46 CET

Oggetto: PLOS ONE Decision: Revision required [PONE-D-19-24032] - [EMID:1be9306ff293a376]

PONE-D-19-24032

Validity of cerebrovascular ICD-9-CM codes in healthcare administrative databases. The Umbria Data-Value Project

PLOS ONE

Dear Dr. Abraha,

Thank you for submitting your manuscript to PLOS ONE. After careful consideration, we feel that it has merit but does not fully meet PLOS ONE’s publication criteria as it currently stands. Therefore, we invite you to submit a revised version of the manuscript that addresses the points raised during the review process.

This study is well conducted. The authors should highlight what is their incremental contribution in terms of methodology to previous studies.

We would appreciate receiving your revised manuscript by Dec 21 2019 11:59PM. When you are ready to submit your revision, log on to https://www.editorialmanager.com/pone/ and select the 'Submissions Needing Revision' folder to locate your manuscript file.

If you would like to make changes to your financial disclosure, please include your updated statement in your cover letter.

To enhance the reproducibility of your results, we recommend that if applicable you deposit your laboratory protocols in protocols.io, where a protocol can be assigned its own identifier (DOI) such that it can be cited independently in the future. For instructions see: http://journals.plos.org/plosone/s/submission-guidelines#loc-laboratory-protocols

Please include the following items when submitting your revised manuscript:

A rebuttal letter that responds to each point raised by the academic editor and reviewer(s). This letter should be uploaded as separate file and labeled 'Response to Reviewers'.

A marked-up copy of your manuscript that highlights changes made to the original version. This file should be uploaded as separate file and labeled 'Revised Manuscript with Track Changes'.

An unmarked version of your revised paper without tracked changes. This file should be uploaded as separate file and labeled 'Manuscript'.

Please note while forming your response, if your article is accepted, you may have the opportunity to make the peer review history publicly available. The record will include editor decision letters (with reviews) and your responses to reviewer comments. If eligible, we will contact you to opt in or out.

We look forward to receiving your revised manuscript.

Kind regards,

Gianni Virgili

Academic Editor

PLOS ONE

Journal Requirements:

1. When submitting your revision, we need you to address these additional requirements.

Please ensure that your manuscript meets PLOS ONE's style requirements, including those for file naming. The PLOS ONE style templates can be found at

http://www.journals.plos.org/plosone/s/file?id=wjVg/PLOSOne_formatting_sample_main_body.pdf and http://www.journals.plos.org/plosone/s/file?id=ba62/PLOSOne_formatting_sample_title_authors_affiliations.pdf

2. During our internal evaluation of the manuscript, we found significant text overlap between your submission and the following previously published works, on which you are an author:

https://journals.plos.org/plosone/article?id=10.1371/journal.pone.0218919

We recognize that the publication cited above was written by you and/or your co-authors. However, please note that re-use of text from a previous publication is unacceptable according to PLOS ONE’s editorial policy on text overlap and re-use (http://journals.plos.org/plosone/s/ethical-publishing-practice#loc-plagiarism). Although this previously published article is Open Access, we still ask that you acknowledge the reuse of any text or data, citing your previous article, so as to properly attribute the original published source. We thank you for your attention to our editorial policies.

Authors’ reply: We modified most of the text overlapping with our previous publication.

Additional Editor Comments (as further peer-review):

The manuscript is very clearly written. The study is well designed with an appropriate choice of controls and adequate validation methods.

Authors’ reply: We thank the Editor for this comment.

The Discussion presents details on previous large systematic reviews on this topic, which encompass a larger time span and include studies using different methodologies. One of these reviews also provides subgroups analyses, which are also presented. The authors should highlight what is the incremental value of their methodology with respect to previous research that could be used in future research and in practice.

Authors’ reply: We added a paragraph in the Discussion section describing what our study adds to the existing literature in the field, with a focus on a comparison with other Italian validation studies.

As a minor comment, avoid presenting numbers (accuracy estimates) in the Abstract conclusions.

Authors’ reply: Thanks for your suggestion. We have deleted the accuracy estimates from the Abstract conclusions.

[Note: HTML markup is below. Please do not edit.]

Reviewers' comments:

Reviewer's Responses to Questions

Comments to the Author

1. Is the manuscript technically sound, and do the data support the conclusions?

The manuscript must describe a technically sound piece of scientific research with data that supports the conclusions. Experiments must have been conducted rigorously, with appropriate controls, replication, and sample sizes. The conclusions must be drawn appropriately based on the data presented.

Reviewer #1: Yes

2. Has the statistical analysis been performed appropriately and rigorously?

Reviewer #1: Yes

3. Have the authors made all data underlying the findings in their manuscript fully available?

The PLOS Data policy requires authors to make all data underlying the findings described in their manuscript fully available without restriction, with rare exception (please refer to the Data Availability Statement in the manuscript PDF file). The data should be provided as part of the manuscript or its supporting information, or deposited to a public repository. For example, in addition to summary statistics, the data points behind means, medians and variance measures should be available. If there are restrictions on publicly sharing data—e.g. participant privacy or use of data from a third party—those must be specified.

Reviewer #1: Yes

4. Is the manuscript presented in an intelligible fashion and written in standard English?

PLOS ONE does not copyedit accepted manuscripts, so the language in submitted articles must be clear, correct, and unambiguous. Any typographical or grammatical errors should be corrected at revision, so please note any specific errors here.

Reviewer #1: Yes

5. Review Comments to the Author

Please use the space provided to explain your answers to the questions above. You may also include additional comments for the author, including concerns about dual publication, research ethics, or publication ethics. (Please upload your review as an attachment if it exceeds 20,000 characters)

**Reviewer #1**: This research article focused on validation of ICD-9 codes for several cerebrovascular conditions in the Umbria regional administrative database in order to use them for future epidemiological studies.

The following MAJOR ISSUES should be carefully considered by the authors:

1) The Authors created several group of diagnoses and demographic and clinical characteristics of patients were described. I suggest adding a new table with all cases in order to describe characteristics of general cohort.

Authors’ reply: We added a new table (Table 1) with a summary of the characteristics of the overall sample.

2) In the discussion section authors reported what is already available in the literature. However, **I miss what this specific study adds to the topic**. I suggest to the authors **to stress the key-message of the study also in the discussion section**.

Authors’ reply: We added a paragraph in the Discussion section describing what our study adds to the existing literature in the field, with a focus on a comparison with other Italian validation studies.

3) The authors declared that they focus on ICD9 diagnosis in primary position. What about the same diagnosis in the second position? Are difference in the number of cases and validity of codes?

Authors’ reply: We decided to limit our validation study to only consider the codes in primary position because, according to the Italian legislation, the primary diagnosis constitutes the main cause of the need for treatment and/or diagnostic tests, and is mainly responsible for the use of resources.

We acknowledged this in the study limitations: “*We acknowledge that a potential limitation of our study is that we did not evaluate the accuracy of ICD-9 codes located in secondary position*”.

6. PLOS authors have the option to publish the peer review history of their article (what does this mean?). If published, this will include your full peer review and any attached files.

If you choose “no”, your identity will remain anonymous but your review may still be made public.

Do you want your identity to be public for this peer review? For information about this choice, including consent withdrawal, please see our Privacy Policy.

Reviewer #1: No

[NOTE: If reviewer comments were submitted as an attachment file, they will be attached to this email and accessible via the submission site. Please log into your account, locate the manuscript record, and check for the action link "View Attachments". If this link does not appear, there are no attachment files to be viewed.]

While revising your submission, please upload your figure files to the Preflight Analysis and Conversion Engine (PACE) digital diagnostic tool, https://pacev2.apexcovantage.com/. PACE helps ensure that figures meet PLOS requirements. To use PACE, you must first register as a user. Registration is free. Then, login and navigate to the UPLOAD tab, where you will find detailed instructions on how to use the tool. If you encounter any issues or have any questions when using PACE, please email us at figures@plos.org. Please note that Supporting Information files do not need this step.
